# WOX11-LBD16 double partner dancing: a feedback circuitry for crown root development in rice

**DOI:** 10.1093/plcell/koae018

**Published:** 2024-01-18

**Authors:** Min-Yao Jhu, Anindya Kundu

**Affiliations:** Assistant Features Editor, The Plant Cell, American Society of Plant Biologists; Crop Science Centre, Department of Plant Sciences, University of Cambridge, Cambridge CB30LE, UK; NIAB, Cambridge CB30LE, UK

In cereals, the primary root degenerates after germination, but adventitious roots, also known as crown roots (CRs), persist throughout the plant’s life cycle. CRs emerge from underground shoot nodes and constitute the primary elements of the root system, serving crucial functions in nutrient absorption, stress resilience, and overall yield (Rogers and Benfey 2015). The regulation of CR development in rice involves WUSCHEL-related homeobox (WOX) and lateral organ boundaries domain (LBD) transcription factors. Yet, the precise molecular mechanisms governing these regulatory processes in rice remain elusive.

**Leping Geng and colleagues** (Geng et al. 2024) unraveled the intricate negative feedback regulatory mechanism of the WOX11-LBD16 module. WOX11 recruits histone demethylase JMJ706 to remove histone H3 lysine 9 dimethylation (H3K9me2) from the *LBD16* promoter, leading to the activation of *LBD16* transcription (Fig.). As LBD16 accumulates, LBD16 competes with JMJ706 for interaction with WOX11, suppressing its own expression by disrupting the WOX11-JMJ706 complex (Fig.). LBD16 facilitates the initiation and elongation of rice CR by modulating cell division. These findings unveil an epigenetic-based feedback control system regulating the expression of genes coordinating rice CR development, with LBD16 serving as a molecular rheostat. This regulatory mechanism strategically fine-tunes *LBD16* expression, preventing an excess of CR growth that could impede overall plant productivity.

**Figure.**
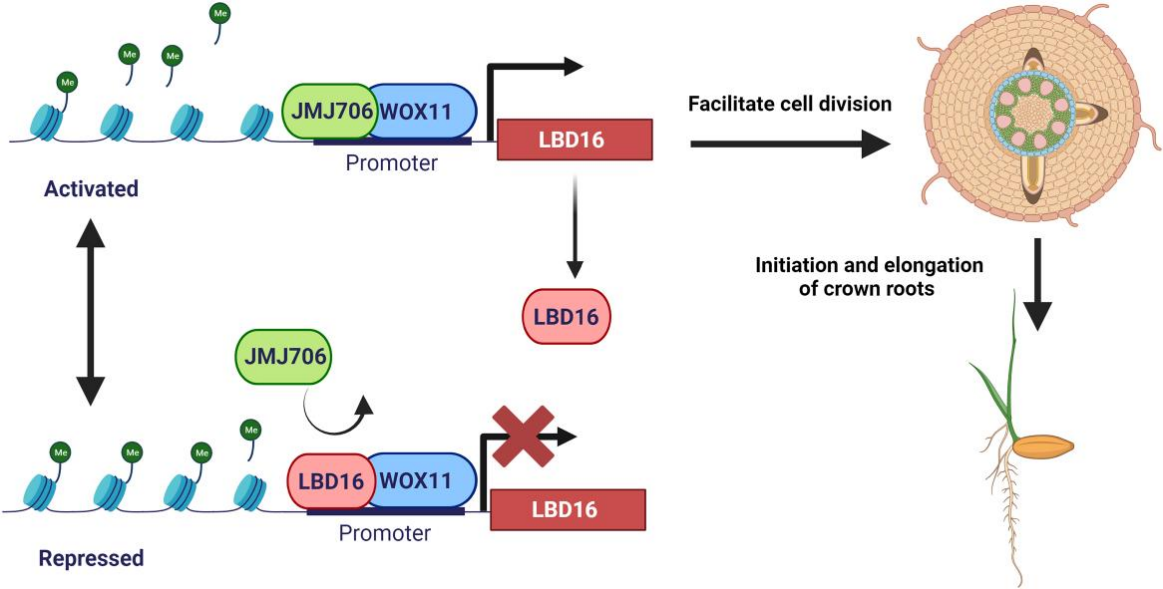
Proposed model of the JMJ706-WOX11-LBD16 module regulating rice crown root development. WOX11 interacts with JMJ706, enabling the removal of H3K9me2 from the LBD16 promoter region, thereby activating LBD16 transcription. Subsequently, increased levels of LBD16 proteins interact with WOX11, displacing JMJ706 and disrupting the activation of LBD16 by the WOX11-JMJ706 complex. The figure was created using BioRender.com, based on the model from Figure 7 in Geng et al. (2024).

While this study identifies the essential contribution of LBD16, it raises intriguing questions about potential interactions between LBD16 and other LBD proteins, such as CROWN ROOTLESS1 (CRL1). Unraveling these dynamics could provide further insights into the comprehensive regulatory network underlying rice root architecture. Furthermore, previous findings demonstrated that WOX11 engages the GCN5-ADA2 complex for histone acetylation, enhancing the expression of metabolic and cell division genes in CRs (Zhou et al. 2017). The current data extend this understanding by revealing WOX11’s recruitment of JMJ706 to demethylate H3K9me2 from the *LBD16* promoter. This dual recruitment of activating histone acetyltransferase GCN5-ADA2 and histone demethylase JMJ706 showcases WOX11’s role as a pioneer transcription factor—a special class of TFs that directly binds condensed chromatin—in this case, initiating chromatin modifications essential for CR initiation and elongation. The interaction between WOX11 and JMJ706 suggests the potential recruitment of other demethylases to downstream target genes. The precise mechanisms linking WOX11 recruitment of JMJ706 and potential impacts on DNA methylation related to cell division remain unclear and warrant further investigation.

Geng et al.’s research sheds light on the functional interplay between WOX11 and LBD16, emphasizing their collaborative role in the intricate dance of CR development. Drawing parallels to observations in *Arabidopsis* (*A. thaliana*), AtWOX11 triggers *AtLBD16* expression in the initiation of axillary roots and callus formation mediated by auxin signaling (Sheng et al. 2017). This evolutionary consistency implies that the WOX11-LBD16 duo plays a pivotal and conserved role in the formation of axillary roots or CR across diverse plant species. However, the absence of any discernible root phenotype upon *LBD16* overexpression in rice, in contrast to numerous primordia observed in *Medicago truncatula* (Schiessl et al. 2019), raises intriguing questions about the specific mechanisms in eudicot and monocot root development.

In conclusion, this research expands our understanding of rice CR development and opens avenues for exploring similar epigenetic mechanisms in other plant developmental processes. The identification of a regulatory network involving WOX11, LBD16, and JMJ706 unveils a captivating molecular choreography that governs the delicate balance between root and shoot growth. Although LBD16 is recruited for root organ development across various species, its epigenetic regulon remains unexplored. Elucidating these complex epigenetic dynamics of *LBD16* expression will allow plants to strike the right balance in root-shoot development, which is crucial for optimizing crop productivity. Geng et al.’s findings provide a valuable molecular toolkit for crop improvement, enhancing root systems through heritable changes without altering the DNA code, and preserving overall plant health and yield.

## Dive Curated Terms

The following phenotypic, genotypic, and functional terms are of significance to the work described in this paper:

JMJ706 Gramene: LOC_Os10g42690
JMJ706 Araport: LOC_Os10g42690
